# Differentiation between G3 pancreatic neuroendocrine tumor and pancreatic neuroendocrine carcinoma based on intratumor and peritumor CT value ratio and abnormal vascular network

**DOI:** 10.3389/fonc.2025.1616763

**Published:** 2025-10-28

**Authors:** Chaoyang Zhang, Wei Hao

**Affiliations:** Department of Radiology, Jincheng General Hospital, Jincheng, Shanxi, China

**Keywords:** pancreatic neuroendocrine tumors, pancreatic neuroendocrine carcinoma, computed tomography, differential diagnosis, prediction model

## Abstract

**Background:**

To develop a model based on computed tomography (CT) images to differentiate between grade 3 (G3) pancreatic neuroendocrine tumors (pNETs) and pancreatic neuroendocrine carcinoma (pNECs).

**Methods:**

This retrospective study included patients with pathologically confirmed pNETs and pNECs who underwent abdominal CT examinations at JINCHENG GENERAL Hospital between June 2012 and June 2023. Tumor and peri-tumor CT characteristics were assessed, including peri-tumor areas A (0–10 mm) and B (10–20 mm) during the arterial and portal venous phases of dynamic enhancement. A model was established using binary logistic regression and receiver operating characteristic (ROC) curves.

**Results:**

A total of 42 patients were included: 20 with G3 pNETs and 22 with pNECs. The ROC analysis showed that the combination of the arterial phase CT ratio B1, portal venous phase CT ratio B2, pancreatic duct invasion (PDI), peripancreatic fat infiltration (PFI), and abnormal vascular network (AVN) [area under the ROC curve (AUC)=0.970 (95% confidence interval (CI): 0.927-1.000), sensitivity=95.50%, and specificity=90.00%] exhibited a better performance in identifying G3 pNETs and pNECs than the combination of the arterial phase CT ratio B1 and the portal venous phase CT ratio B2 [AUC = 0.907 (95% CI: 0.818-0.996), sensitivity=77.30%, and specificity=95.00%], and the combination of arterial phase CT ratio B1, portal venous phase CT ratio B2, and AVN [AUC = 0.923 (95% CI: 0.810-1.000), sensitivity=81.80%, and specificity=85.00%].

**Conclusion:**

The enhancement ratio between the tumor and peri-tumoral B area in the arterial and portal venous phases, along with AVN, PFI, and PDI, may serve as effective indicators for distinguishing pNECs from G3 pNETs.

## Introduction

Pancreatic neuroendocrine neoplasms (pNENs) exhibit significant heterogeneity and various biological characteristics (1). The different hormones secreted by pNENs can lead to a wide variety of clinical symptoms among patients (2, 3). Low-grade pNENs often display an indolent growth pattern, whereas high-grade pNENs are invasive and tend to metastasize early (4). In recent years, the incidence of pNENs has significantly increased due to advancements in examination technology, allowing the detection of smaller tumors (5). According to the World Health Organization (WHO) 2019 classification standards, pNENs can be classified into grade G1-G3-grade pancreatic neuroendocrine tumors (pNETs) and pancreatic neuroendocrine carcinomas (pNECs) (6). G3 pNETs and pNECs are distinct entities with overlapping features that complicate diagnosis, yet differ in pathology (7–10), treatment strategies (7–10), and prognosis (11–15). G3 pNETs may benefit from therapies used for lower-grade NETs (alkylating agents, somatostatin analogs, targeted therapies), and responses to platinum-based chemotherapy are generally poor (16–19). pNECs, conversely, show greater response to platinum-based regimens (cisplatin/etoposide) typical for high-grade carcinomas (16, 20, 21). Hence, the precise differentiation between G3 pNETs and pNECs is crucial.

Still, differentiating between G3 pNETs and pNECs is not easy. Pathologically, Distinguishing G3 pNETs from pNECs is difficult due to shared proliferation indices (Ki67 > 20%), leading to diagnostic ambiguity in some cases despite immunohistochemistry and molecular testing (22), and more aggressive histopathological features (23), but biopsies can be unreliable since they sample only a small part of the tumor that can be unrepresentative of the whole tumor, and waiting for surgery and the pathological examination of the specimen can lead to missed therapeutic opportunities (22). Ideally, the early differentiation between pNETs and pNECs should be based on imaging since it is non-invasive and can be performed early in the course of the disease. Magnetic resonance imaging (MRI) can differentiate between pNETs and pNECs (24), but MRI has accessibility, availability, and cost issues, particularly in developing countries. Computed tomography (CT) is the first-line imaging modality in patients suspected of pNENs, but it has a lower resolution than MRI for soft tissues (25). CT-based models have been developed successfully for the differential diagnosis of pNETs and pancreatic duct adenocarcinoma (PDAC), either using age, pancreatic duct dilatation, and portal enhancement ratio (26), or CA 19–9 levels, tumor shape, and pancreatic duct dilation (27), but pNECs were not included, while another model (tumor margin, enhanced degree, parenchymal atrophy, and texture parameters) could differentiate between pNECs and PDAC but did not include pNETs (28). Two studies in pNENs showed that some CT texture features could differentiate different tumor types (29, 30). Nevertheless, pNENs are rare tumors, and a CT-based model differentiating between G3 pNETs and pNECs is lacking.

It was hypothesized that pNETs and pNECs display significant differences in the relationships among the tumor, pancreatic duct, and peripancreatic fat, the abnormal vascular network (AVN) surrounding the tumor, and the enhancement ratio between tumor and normal tissues. This study aimed to determine a model based on CT images to differentiate between pNETs and pNECs based on those features. The results could help improve the diagnosis of pNETs vs. pNECs and patient management.

## Materials and methods

### Study design and patients

This retrospective study collected patients with pNEN who underwent abdominal CT examination at the JINCHENG GENERAL Hospital between June 2012 and June 2023. This study was approved by the ethics committee of the JINCHENG GENERAL Hospital (approval #LL2024032101). The requirement for individual consent was waived due to the retrospective nature of the study. The inclusion criteria were 1) pathological diagnosis of G3 grade PNET or PNEC, 2) did not receive any anti-tumor treatment before abdominal CT examination, 3) CT image quality was satisfactory, clearly displaying the lesion and surrounding areas, and 4) complete medical data. The exclusion criteria were 1) the CT images contained severe artifacts or noise that affected measurement, or 2) the biopsy or surgical pathology results indicated G1 or G2 pNET.

### Data collection and definitions

#### Image acquisition

During the entire study period, an Optima 680 CT instrument (GE Healthcare, Waukesha, WI, USA) was used to conduct a thorough scan of the abdomen, ranging from the top of the diaphragm to the level of the renal hilum. The routine parameters were tube voltage of 120 kV, tube current of 250 mA, pitch of 0.992, field of view of 360 mm, matrix of 512×512, and layer thickness and layer spacing of 3 mm. After the initial scan, 100 mL of the contrast agent (iopromide, 240 mgI/ml) was injected through the cubital vein at a flow rate of 2.5 mL/s. When the CT value of the abdominal aorta reached 100 HU, the arterial phase scan was initiated automatically, and the portal vein scan was conducted 70 s later.

### Image analysis

Two experienced radiologists used the Picture Archiving and Communication System (PACS) to analyze and interpret the images. In case of disagreement, a decision was reached through discussion and agreement. Tumor size, tumor location, tumor composition, calcification, pancreatic duct invasion (PDI), peripancreatic fat infiltration (PFI), and AVN were determined. PDI was defined as the distal expansion of the main pancreatic duct near the lesion. PFI was defined as the peripancreatic fat being unclear and not smooth. AVN was defined as an abnormally proliferating vascular network surrounding the tumor.

When measuring the CT values, the tumor body and two specific areas around the tumor (A and B zones) were included, while the cystic necrosis regions in the tumor body and the non-pancreatic tissues were excluded. The tumor area was manually delineated, and zones A and B were automatically delineated by the CT system. The CT value of necrotic/cystic areas within the tumor generally ranges from 10 to 20 HU, and they do not enhance during any phase of enhancement. The first area, called peritumoral A, refers to the region between the tumor edge and a distance of 10 mm surrounding it. The second area, referred to as peritumoral B, extends from 10 to 20 mm surrounding the tumor edge, excluding non-pancreatic tissues. A region of interest (ROI) with an area of 10 mm^2^ was manually outlined, and the CT values of the tumor and peritumoral areas were measured in the arterial and portal venous phases. These values were measured thrice to calculate the average. The tumor and portal venous phase values were finally calculated in the arterial and portal venous phases, respectively. The enhancement ratio of the peritumoral A area (CT ratio A1, A2) and the enhancement ratio of the tumor body to the peritumoral B area (CT ratio B1, B2) were also determined as follows. CT ratio A1=arterial phase A-zone CT value ÷ arterial phase tumor CT value. CT ratio B1=arterial phase B-zone CT value ÷ arterial phase tumor CT value. CT ratio A2=venous phase A-zone CT value ÷ venous phase tumor CT value. CT ratio B2=venous phase B-zone CT value ÷ venous phase tumor CT value.

### Statistical analysis

SPSS 25.0 (IBM, Armonk, NY, USA) was used to perform the statistical analysis. The continuous data following a normal distribution were expressed as means ± standard deviations and analyzed using Student’s t-test. The categorical data were presented as n (%) and analyzed using the chi-squared test or Fisher’s exact probability test. The continuous and categorical data were analyzed using univariable logistic regression to identify useful indicators for distinguishing G3 grade pNETs and pNECs. A multivariable binary logistic regression model was constructed to evaluate its discriminant performance. The variables with P<0.05 in the univariable analyses and those with clinical significance were included in the multivariable binary logistic regression analysis (enter method). A receiver operating characteristic (ROC) curve analysis was performed to determine the performance of the model, based on the area under the curve (AUC). An agreement test was performed to determine the intraclass coefficient (ICC) between the two radiologists. Two-sided P-values <0.05 were considered statistically significant.

## Results

### Characteristics of the patients

**Figure 1:**
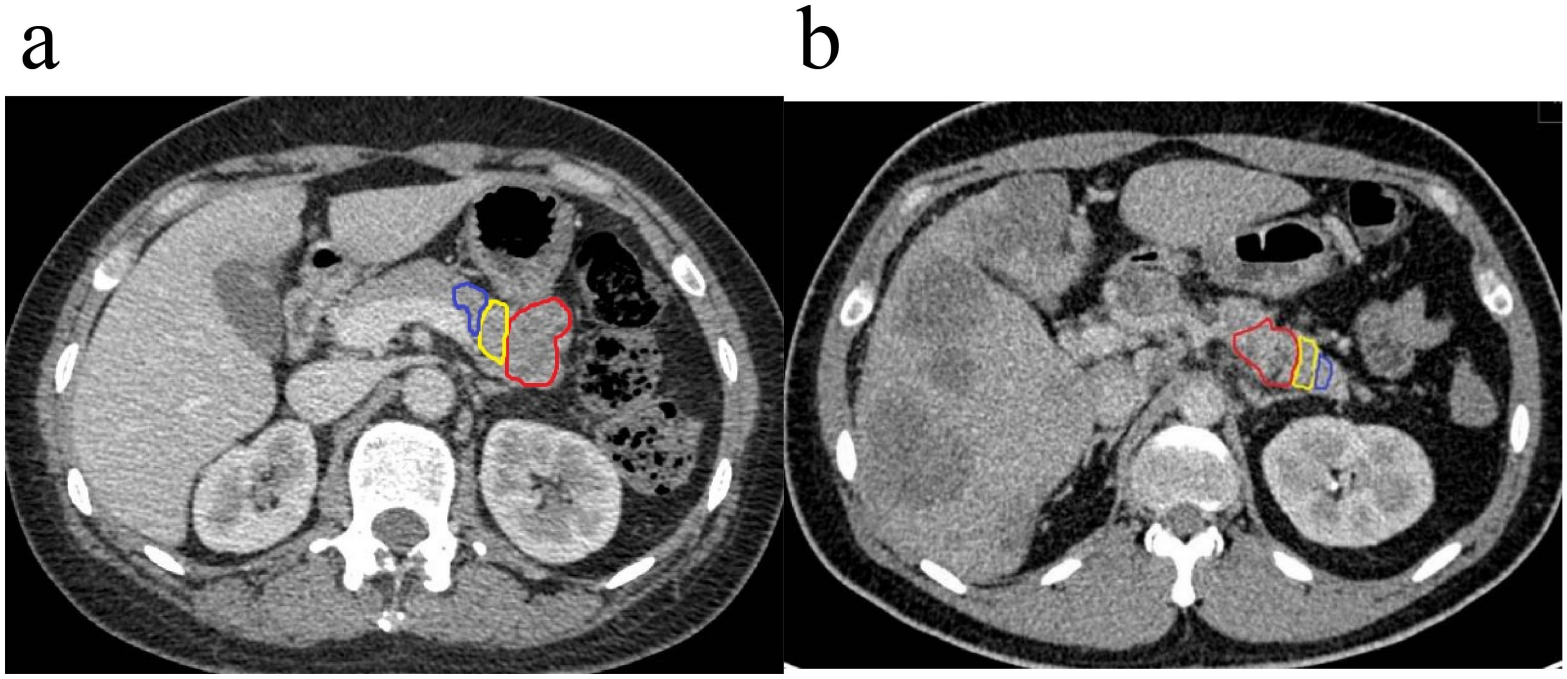
Region of interest (ROI) delineated in axial portal phase computed tomography (CT). **(a)** A 63-year-old male patient was diagnosed with G3 pancreatic neuroendocrine tumor (pNET) at the tail of the pancreas, and the space between the adjacent gastric body fat had disappeared. **(b)** A 34-year-old male patient presented with pancreatic neuroendocrine carcinoma (pNEC) in the pancreatic body, with the tumor invading the nearby splenic artery, showing rough and localized narrowing, multiple intrahepatic metastases, and peritoneum multiple enlarged lymph nodes (the red area is the tumor body, the yellow area is peritumor **(a)** the blue area is peritumor **(b)**.

The 42 patients included 21 males and 21 females, aged between 27 and 75 years, with an average age of 54.74 ± 12.10. There were 20 patients in the G3 pNET group and 22 patients in the pNEC group. In the G3 pNET group, there were 12 males and eight females, ranging in age from 27 to 73 years, with an average age of 53.65 ± 13.39 years. In the pNEC group, there were nine males and 13 females, ranging in age from 32 to 75 years, with an average age of 55.73 ± 11.00 years. There were no significant differences in age, sex, tumor size, calcification, composition, arterial phase, and portal venous phase A area enhancement ratio (CT ratio A1, A2) between the two groups (all P>0.05). However, there were statistically significant differences in PDI, PFI, AVN, and arterial and portal venous phase enhancement ratios (CT ratios B1 and B2) (all P<0.05) (**Table 1**, **Figure 1**).

**Table 1:** Comparison of the general information and CT characteristics between the G3 grade NET group and NEC group.

Features	G3 NET (n=20)	NEC (n=22)	t/χ*^2^*	P
Age, years	53.65 ± 13.39	55.73 ± 11.00	-0.551	0.585
Sex, M (F)	12 (8)	9 (13)	1.527	0.354
Size, mm	55.20 ± 45.67	44.0 ± 26.0	0.941	0.352
Solid (cystic)	20 (0)	18 (4)	4.019	0.109
Calcification	1	2	0.264	0.607
PFI	7	17	7.644	<0.05
PDI	7	16	6.019	<0.05
AVN	2	14	12.780	0.001
Arterial phase				
CT ratio A1	0.72 ± 0.10	0.71 ± 0.10	0.276	0.784
CT ratio B1	0.79± 0.12	0.68± 0.08	3.470	0.001
Venous Phase				
CT ratio A2	0.83± 0.07	0.79± 0.09	1.601	0.117
CT ratio B2	1.05 ± 0.10	0.88 ± 0.10	5.487	<0.001

G3 NET, grade 3 neuroendocrine tumor; NEC, neuroendocrine carcinoma; M, male; F, female; PFI, peripancreatic fat infiltration; PDI, pancreatic duct invasion; AVN, abnormal vascular network; CT, computed tomography.

In addition, the agreement test showed that the CT value of the tumor in arterial stage (ICC = 0.946), peritumor area A in arterial stage (ICC = 0.983), peritumor B area in arterial stage (ICC = 0.989), tumor in venous stage (ICC = 0.979), peritumor area A in venous stage (ICC = 0.986), and peritumor B area in venous stage (ICC = 0.979) examined by the two radiologists had a favorable consistency (**Supplementary Table S1**). The overall conformity was ICC = 0.951.

### Constructing the model

**Figure 2:**
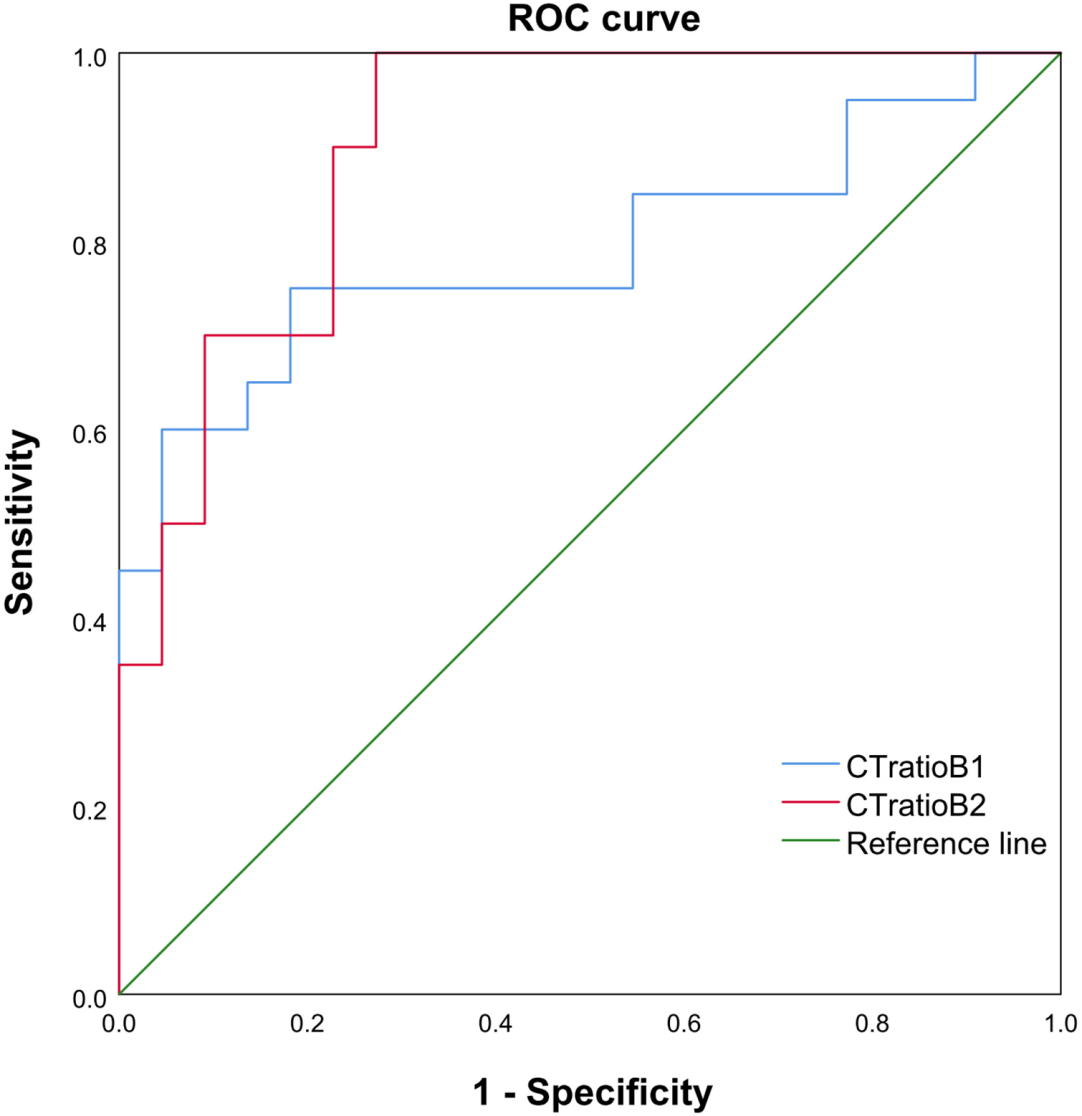
The receiver operating characteristics (ROC) curve, cut point value, and corresponding sensitivity as well as specificity of each cut point value of CT ratio B1 and CT ratio B2 between pNECs and G3 grade (pNETs). The areas under the curve (AUCs) for CT ratio B1 and B2 were 0.791 (95% CI: 0.645, 0.937) and 0.902 (95% CI: 0.812, 0.993), respectively. The sensitivity and specificity were found to be the highest for CT ratio B2 <0.914.

The univariable logistic regression analyses showed that PDI, PFI, AVN, and portal venous phase CT ratios could be used to differentiate G3 pNETs from pNECs (**Tables 2**, **3**). Patients in the NEC group exhibited a higher frequency of tumors with PDI (odds ratio (OR)=2.203, 95% confidence interval (CI): 1.078-4.505), PFI (OR = 2.551, 95%CI: 1.160-5.618), and AVN (OR = 2.841, 95%CI: 1.395-5.208) (**Table 2**). The AUCs of arterial phase and portal venous phase CT ratios for distinguishing G3 pNETs from pNECs were 0.791 (95%CI: 0.645-0.937) and 0.902 (95%CI: 0.812-0.993), respectively. When the arterial phase CT ratio cutoff value was 0.759, the sensitivity and specificity were 75.00% and 81.80%, respectively. When the portal venous phase CT ratio cutoff value was 0.914, the sensitivity and specificity were 95.00% and 72.70%, respectively (**Table 3**, **Figure 2**).

**Table 2:** Univariate logistic regression analysis of G3 grade NET group and NEC group 1 (*n* = 42).

Features	β	SE	P	OR	95%CI
PDI	1.949	0.892	<0.05	2.203	1.078-4.505
PFI	1.624	0.884	<0.05	2.551	1.160-5.618
AVN	2.739	1.089	<0.05	2.841	1.395-5.208

SE, standard error; OR, odds ratio; CI, confidence interval; PDI, pancreatic duct invasion; PFI, peripancreatic fat infiltration; AVN, abnormal vascular network.

**Table 3:** Univariate logistic regression analysis of G3 grade NET group and NEC group 2 (*n* = 42).

CT ratio	AUC	Sensitivity	Specificity	Cut point
CT ratio B1	0.791 (95%CI: 0.645, 0.937)	75.00%	81.80%	0.759
CT ratio B2	0.902 (95%CI: 0.812, 0.993)	95.00%	72.70%	0.914

AUC, area under the curve; CT, computed tomography; CI, confidence interval.

**Figure 3:**
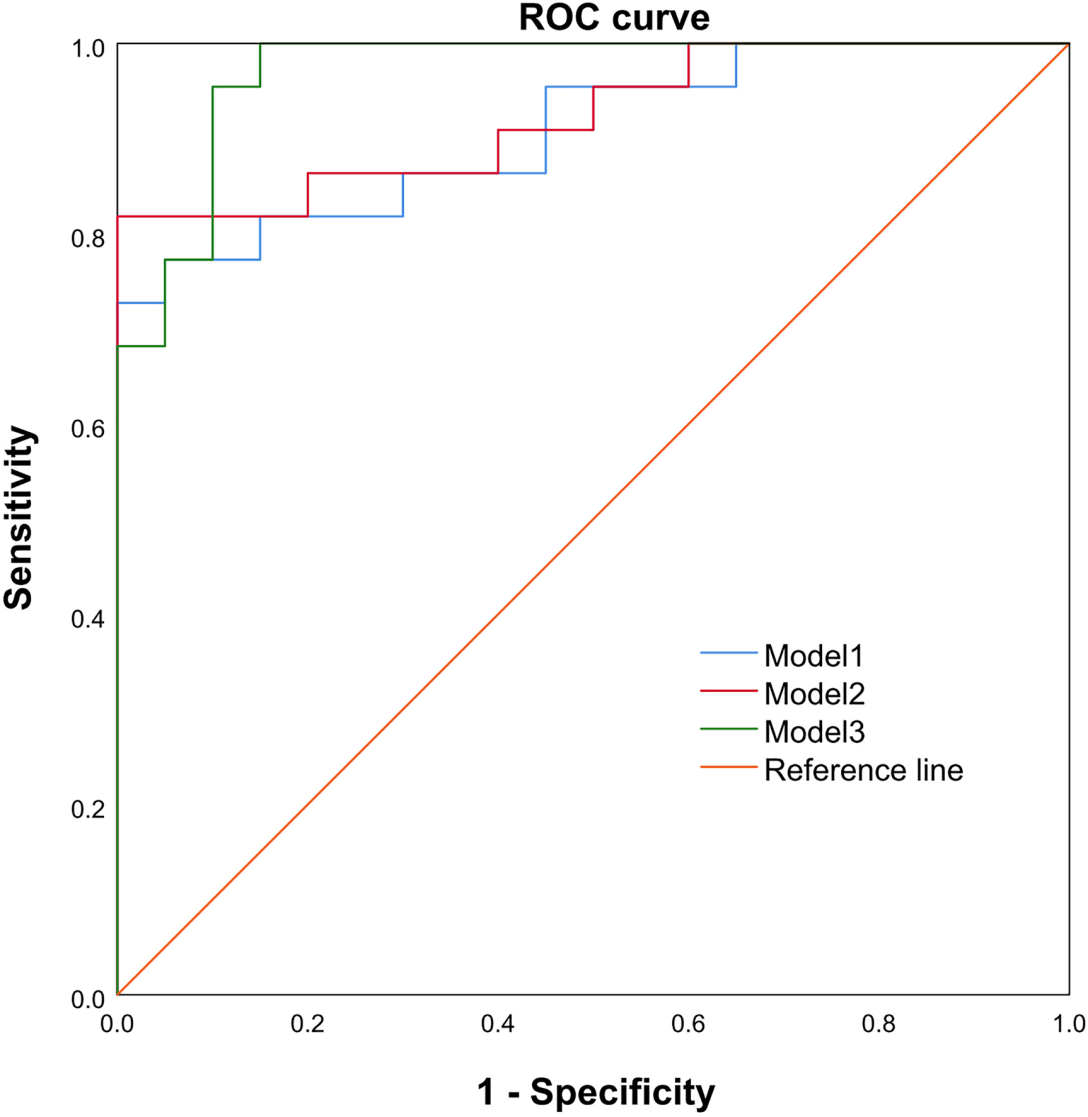
The ROC curve, cut point value, and corresponding sensitivity, as well as the specificity of each cut point value of Models 1, 2, and 3 between pNEC and G3 grade pNET. The AUCs for Models 1, 2, and 3 were 0.907 (95%CI: 0.818, 0.996), 0.923 (95%CI: 0.841, 1.000), and 0.970 (95%CI: 0.927-1.000), 95.50% and 90.00%, respectively. The sensitivity and specificity were the highest for Model 3.

The ROC analysis showed that the combination of the arterial phase CT ratio B1, portal venous phase CT ratio B2, PDI, PFI, and AVN (AUC = 0.970, 95%CI: 0.927-1.000, sensitivity of 95.50%, and specificity of 90.00%) exhibited a better performance in identifying G3 pNETs and pNECs than the combination of the arterial phase CT ratio B1 and the portal venous phase CT ratio B2 (AUC = 0.907, 95%CI: 0.818-0.996, sensitivity of 77.30%, and specificity of 95.00%), and the combination of arterial phase CT ratio B1, portal venous phase CT ratio B2, and AVN (AUC = 0.923, 95% CI: 0.810-1.000, sensitivity of 81.80%, and specificity of 85.00%) (**Table 4**, **Figure 3**).

**Table 4:** Multivariable binary logistic regression analysis of G3 grade NET group and NEC group (*n* = 42).

Combination Model	AUC	Sensitivity	Specificity	Cut point
Model 1	0.907 (95%CI: 0.818, 0.996)	77.30%	95.00%	0.735
Model 2	0.923 (95%CI: 0.841, 1.000)	81.80%	85.00%	0.683
Model 3	0.970 (95%CI: 0.927, 1.000)	95.50%	90.00%	0.432

AUC, area under the curve; CT, computed tomography; CI, confidence interval; PFI, peripancreatic fat infiltration; PDI, pancreatic duct invasion; AVN, abnormal vascular network; CT, computed tomography.

Model 1, arterial phase CT ratio B1 and portal venous phase CT ratio B2.

Model 2, arterial phase CT ratio B1, portal venous phase CT ratio B2, and AVN.

Model 3, arterial phase CT ratio B1, portal venous phase CT ratio B2, PDI, PFI, and AVN.

## Discussion

This retrospective study built models based on CT images to differentiate between G3 pNETs and pNECs. The results suggested that the enhancement ratio between the tumor and peritumoral B area in the arterial and portal venous phases, AVN, PFI, and PDI, could serve as an indicator for effectively distinguishing pNECs from G3 pNETs.

The present study revealed no statistically significant differences between patients with G3 pNETs vs. pNECs in terms of demographic (age and sex), histological (tumor size and calcification), and imaging (composition, arterial phase, and portal venous phase CT ratio A) characteristics, consistent with the study by Belousova et al. (31).

The present study employed a diagnostic approach combining quantitative and qualitative analysis. By combining the arterial phase CT ratio B1 and the portal venous phase CT ratio B2, the study aimed to detect intra-pancreatic PDI, PFI, and AVN. These factors were combined to establish a multivariable binary logistic regression model to develop a reliable method for distinguishing between pNECs and G3 pNETs.

Recent studies indicated that distinguishing between the two can be effectively accomplished by considering the arterial and portal venous phases of CT ratios B1 and B2, which have been identified as autonomous predictive factors (29, 30). It could be because higher-grade PNETs have lower tumor angiogenesis than lower-grade PNETs, consistent with the WHO 2017 classification system (32, 33). A previous study on the grading of pNETs was consistent with the WHO 2019 classification system and pathological analysis (6) and was similar to two previous studies on arterial and portal vein enhancement rates (34, 35). The previous studies relied on specific cutoff values of arterial and portal vein enhancement rates to identify G1, G2, and G3 pNETs, respectively, and to identify G1, G2, and G3 pNETs and pNECs. However, the measured CT values did not consider the presence of peritumoral edema and adjacent pancreatic tissue. Due to invasion and other factors, the peritumoral area was not divided into different regions, and the inconsistent CT values in different areas surrounding the tumor were not properly analyzed and documented. In this study, the tumor area was finely divided into the peritumoral zone A, closer to the lesion (0–10 mm), and the peritumoral zone B, farther from the lesion (10–20 mm). The CT values of the pancreatic lesion and the adjacent tissue are commonly affected by unstable factors such as peritumoral edema, inflammation, necrosis, and adjacent tissue invasion (36, 37); therefore, the CT value of zone B (i.e., 10-20-mm peritumoral) was measured because it was hypothesized to be less likely affected by peritumoral edema, inflammation, necrosis, and adjacent tissue invasion. The present study considered the fact that the CT value remained relatively stable in the peritumoral B area, located far from the lesion, compared with the tumor body and peritumoral A area, and pNECs and G3 pNETs can be distinguished based on their CT ratio.

Then, based on the univariable logistic regression model, pNECs exhibited a higher propensity for PDI compared with G3 pNETs, as well as higher frequencies of PFI and AVN. G3 pNETs infrequently invade the main pancreatic duct (MPD); when present, intraductal extension is considered a unique and rare growth pattern. On the other hand, pNECs are more aggressive, with rapid progression and a higher frequency of invasion into adjacent tissues, including the ductal system (23, 24, 38). The extent and pattern of PFI on imaging (CT/MRI) help differentiate the more aggressive, poorly differentiated pNEC from G3 pNETs, though overlap exists, especially in advanced tumors (39–42). Both tumor types can infiltrate surrounding fat, but pNECs are characterized by higher grade, poor differentiation, and worse prognosis, resulting in more frequent extensive infiltration beyond the pancreas (39, 40). Regarding AVN, G3 pNETs often preserve a well-organized, organoid vascular network that is characteristic of well-differentiated neuroendocrine cells, including abundant neurosecretory granules and relatively uniform vessel distribution (23, 43). G3 pNECs typically display more chaotic, disorganized, and poorly formed vasculature, reflecting their poorly differentiated cytology and aggressive biological behavior (22, 23, 43). All three features (PDI, PFI, and AVN) can be observed on enhanced CT images.

At present, the diagnosis, identification, and prediction of G3 pNETs and pNECs are based on diverse imaging techniques and methods such as dynamic enhanced CT and MRI gray-scale ratio, histogram analysis, radiomics, deep learning, and transfer learning (44–47). In addition, there is currently an increasing emphasis on assessment. For example, De Robertis et al. (48) analyzed the apparent diffusion coefficient (ADC) value of tumors and used histograms to predict tumor invasiveness. Analyzing pNEN histograms on ADC maps could aid in predicting tumor grade, vascular invasion, lymph nodes, and liver metastases in pNENs. The most precise indicators for detecting pNENs with aggressive tendencies are ADC entropy and ADC kurtosis. They concluded that ADC entropy and ADC peak were the most reliable variables for distinguishing pNENs displaying malignant characteristics. In another study, Li et al. (49) examined radiomics features based on MRI images and could effectively predict the grade of pNETs. Their findings suggested that higher grades of pNETs were associated with increased metastasis risk and unfavorable prognosis. Xu et al. (24) reported that MRI could differentiate between pNETs and pNECs. Indeed, MRI has high sensitivity but has inconveniences like accessibility, availability, and cost, especially in developing countries (50, 51). Hence, CT could be more appropriate than MRI for the workup of pNENs. Nevertheless, limited information exists regarding the application of the observation of the peritumoral vascular network, peritumoral divisions, and CT ratios of the tumor body and peritumoral area during the arterial phase and portal venous phase for the differential diagnosis of G3 pNETs and pNECs. In this study, by examining the various characteristics of the tumor and peritumoral areas, measuring and calculating the average CT value of the ROI in the solid area of ​​the tumor and the peritumoral B area, and the ratio of each phase after enhancement, accurate and dependable indicators were identified for distinguishing between the two tumor types.

### Strengths and limitations

A strength of this study is the use of CT images that were consistently obtained using the same scanner among all patients and analyzed uniformly by the same two radiologists. Still, this study has limitations. The study was performed at a single center, and the sample size was small since G3 pNETs and pNECs are relatively rare tumors. Still, the sample size in the present study compares with previous ones (29, 35, 49). The study was retrospective and limited to the images and data found in the patient charts. Although a model was successfully built and displayed a high AUC (0.970), no external validation was performed. There is no widely accepted anatomical or biological justification in the literature for dividing pancreatic peritumoral zones specifically into 0–10 mm (zone A) and 10–20 mm (zone B); these boundaries are arbitrary and not based on robust anatomical or tumor biology data (52–56). Still, cutoffs had to be determined and tried, and those are the ones tried in the present study. Finally, due to the available data and the small sample size, the model developed here could not be compared against other models based on KI67, histopathology, or MRI. Additional studies are necessary to confirm the study findings.

### Clinical significance

Abdominal enhanced CT is a first-line imaging examination method for pNEN (1, 25–27, 29, 31, 34, 46). In this study, AVN, PDI, and PFI were observed, and the ratio of enhanced CT tumor body to peritumoral CT values was calculated to differentiate G3 grade pNET from pNEC. The AVN, PDI, PFI, and the ratio of arterial/portal venous phase to plain CT values can objectively reflect the nature of the tumor, and the magnitude of their changes was associated with the degree of tissue infiltration around the tumor and the presence of distant metastasis. The CT measurements of pancreatic tumors and the surrounding tissues are frequently influenced by variable factors, including swelling around the tumor, inflammation, tissue death, and infiltration into adjacent structures (36, 37); therefore, the CT value of zone B (i.e., 10-20-mm peritumoral) was measured. The results showed that the CT ratios B1 and B2 in the arterial and portal venous phases were higher in the pNET group than in the pNEC group (P<0.001 for both). Considering that the median survival time of G3 grade pNET patients is 41–99 months, while that of pNEC patients is only 8–13 months (11–15), and considering that the treatment strategies for the two diseases are different (7–10), the clinical significance is that accurately distinguishing G3 grade pNET from pNEC using the commonly-used enhanced CT examination is important in terms of patient management, avoid under- or over-treatment, and prognosis determination.

## Conclusion

The results suggest that the CT ratio of the tumor and peritumoral B area, AVN, PDI, and PFI can be combined into a model to differentiate between G3 pNETs and pNECs with a high diagnostic performance.

## Data Availability

The original contributions presented in the study are included in the article/**Supplementary Material**. Further inquiries can be directed to the corresponding author.
