# Supplementary material for: Differentiation between G3 pancreatic neuroendocrine tumor and pancreatic neuroendocrine carcinoma based on intratumor and peritumor CT value ratio and abnormal vascular network
Source: Front Oncol. 2025 Oct 28;15:1616763. doi: 10.3389/fonc.2025.1616763 (PMC12602241; doi:10.3389/fonc.2025.1616763)
Supplement: Supplementary file 1 [file Table1.docx]

**Supplementary Table S1.** Conformity test.

| Group | Patient Number | CT value of tumor in arterial stage | | CT value of peritumor area A in arterial stage | | CT value of peritumor B area in arterial stage | | CT value of tumor in venous stage | | CT value of peritumor area A in venous stage | | CT value of peritumor B area in venous stage | | |
| --- | --- | --- | --- | --- | --- | --- | --- | --- | --- | --- | --- | --- | --- | --- |
|  |  | Physician 1# | Physician 2# | Physician 1# | Physician 2# | Physician 1# | Physician 2# | Physician 1# | Physician 2# | Physician 1# | Physician 2# | Physician 1# | Physician 2# |  |
| G3 pNET | NO.1 | 74.00 | 76.00 | 100.00 | 102.00 | 94.00 | 96.00 | 88.00 | 90.00 | 108.00 | 106.00 | 89.00 | 89.00 |  |
|  | NO.2 | 88.00 | 90.00 | 125.00 | 127.00 | 100.00 | 102.00 | 97.00 | 100.00 | 114.00 | 116.00 | 87.00 | 90.00 |  |
|  | NO.3 | 90.00 | 92.00 | 101.00 | 103.00 | 114.00 | 117.00 | 95.00 | 97.00 | 108.00 | 108.00 | 107.00 | 107.00 |  |
|  | NO.4 | 70.00 | 72.00 | 112.00 | 113.00 | 105.00 | 107.00 | 81.00 | 83.00 | 109.00 | 107.00 | 87.00 | 93.00 |  |
|  | NO.5 | 72.00 | 75.00 | 100.00 | 102.00 | 87.00 | 89.00 | 71.00 | 73.00 | 100.00 | 102.00 | 75.00 | 77.00 |  |
|  | NO.6 | 101.00 | 101.00 | 113.00 | 115.00 | 97.00 | 99.00 | 104.00 | 106.00 | 125.00 | 127.00 | 86.00 | 88.00 |  |
|  | NO.7 | 80.00 | 82.00 | 100.00 | 102.00 | 95.00 | 97.00 | 88.00 | 90.00 | 98.00 | 102.00 | 81.00 | 77.00 |  |
|  | NO.8 | 71.00 | 73.00 | 103.00 | 101.00 | 93.00 | 95.00 | 88.00 | 90.00 | 97.00 | 98.00 | 87.00 | 88.00 |  |
|  | NO.9 | 101.00 | 100.00 | 109.00 | 112.00 | 98.00 | 99.00 | 96.00 | 98.00 | 116.00 | 113.00 | 86.00 | 84.00 |  |
|  | NO.10 | 76.00 | 70.00 | 98.00 | 95.00 | 85.00 | 83.00 | 88.00 | 90.00 | 87.00 | 85.00 | 71.00 | 73.00 |  |
|  | NO.11 | 62.00 | 60.00 | 96.00 | 99.00 | 89.00 | 91.00 | 75.00 | 72.00 | 81.00 | 86.00 | 78.00 | 79.00 |  |
|  | NO.12 | 70.00 | 72.00 | 109.00 | 111.00 | 92.00 | 90.00 | 78.00 | 80.00 | 99.00 | 97.00 | 77.00 | 79.00 |  |
|  | NO.13 | 78.00 | 82.00 | 105.00 | 103.00 | 95.00 | 95.00 | 84.00 | 88.00 | 95.00 | 97.00 | 82.00 | 80.00 |  |
|  | NO.14 | 89.00 | 91.00 | 125.00 | 128.00 | 108.00 | 112.00 | 117.00 | 119.00 | 132.00 | 130.00 | 101.00 | 99.00 |  |
|  | NO.15 | 71.00 | 69.00 | 94.00 | 98.00 | 87.00 | 89.00 | 75.00 | 77.00 | 78.00 | 76.00 | 78.00 | 82.00 |  |
|  | NO.16 | 76.00 | 78.00 | 104.00 | 102.00 | 89.00 | 91.00 | 80.00 | 78.00 | 92.00 | 90.00 | 80.00 | 85.00 |  |
|  | NO.17 | 68.00 | 70.00 | 96.00 | 98.00 | 91.00 | 89.00 | 78.00 | 80.00 | 111.00 | 110.00 | 78.00 | 82.00 |  |
|  | NO.18 | 55.00 | 59.00 | 99.00 | 97.00 | 96.00 | 98.00 | 80.00 | 76.00 | 89.00 | 87.00 | 77.00 | 75.00 |  |
|  | NO.19 | 66.00 | 68.00 | 102.00 | 100.00 | 93.00 | 95.00 | 83.00 | 81.00 | 108.00 | 112.00 | 80.00 | 78.00 |  |
|  | NO.20 | 74.00 | 76.00 | 103.00 | 101.00 | 96.00 | 94.00 | 84.00 | 82.00 | 106.00 | 104.00 | 85.00 | 87.00 |  |
| pNEC | NO.1 | 72.00 | 74.00 | 83.00 | 81.00 | 91.00 | 89.00 | 59.00 | 61.00 | 69.00 | 67.00 | 69.00 | 71.00 |  |
|  | NO.2 | 71.00 | 57.00 | 95.00 | 93.00 | 103.00 | 101.00 | 84.00 | 82.00 | 103.00 | 101.00 | 88.00 | 86.00 |  |
|  | NO.3 | 66.00 | 68.00 | 76.00 | 78.00 | 88.00 | 90.00 | 77.00 | 79.00 | 86.00 | 84.00 | 69.00 | 71.00 |  |
|  | NO.4 | 68.00 | 66.00 | 97.00 | 99.00 | 87.00 | 83.00 | 74.00 | 76.00 | 96.00 | 94.00 | 75.00 | 77.00 |  |
|  | NO.5 | 80.00 | 78.00 | 94.00 | 92.00 | 105.00 | 107.00 | 86.00 | 84.00 | 92.00 | 90.00 | 96.00 | 100.00 |  |
|  | NO.6 | 65.00 | 65.00 | 86.00 | 86.00 | 89.00 | 88.00 | 73.00 | 75.00 | 86.00 | 87.00 | 71.00 | 69.00 |  |
|  | NO.7 | 62.00 | 64.00 | 114.00 | 116.00 | 91.00 | 93.00 | 73.00 | 75.00 | 102.00 | 98.00 | 84.00 | 80.00 |  |
|  | NO.8 | 80.00 | 78.00 | 115.00 | 119.00 | 134.00 | 138.00 | 88.00 | 90.00 | 110.00 | 108.00 | 98.00 | 96.00 |  |
|  | NO.9 | 70.00 | 68.00 | 97.00 | 98.00 | 117.00 | 119.00 | 97.00 | 99.00 | 112.00 | 114.00 | 107.00 | 109.00 |  |
|  | NO.10 | 86.00 | 82.00 | 123.00 | 127.00 | 108.00 | 106.00 | 95.00 | 97.00 | 101.00 | 103.00 | 98.00 | 96.00 |  |
|  | NO.11 | 75.00 | 79.00 | 132.00 | 130.00 | 137.00 | 139.00 | 77.00 | 79.00 | 109.00 | 111.00 | 120.00 | 122.00 |  |
|  | NO.12 | 77.00 | 74.00 | 105.00 | 107.00 | 111.00 | 109.00 | 81.00 | 83.00 | 108.00 | 110.00 | 95.00 | 93.00 |  |
|  | NO.13 | 71.00 | 73.00 | 96.00 | 98.00 | 111.00 | 109.00 | 79.00 | 81.00 | 117.00 | 119.00 | 90.00 | 88.00 |  |
|  | NO.14 | 60.00 | 63.00 | 119.00 | 121.00 | 107.00 | 109.00 | 71.00 | 73.00 | 95.00 | 97.00 | 81.00 | 83.00 |  |
|  | NO.15 | 77.00 | 75.00 | 120.00 | 122.00 | 147.00 | 145.00 | 79.00 | 81.00 | 111.00 | 109.00 | 95.00 | 97.00 |  |
|  | NO.16 | 61.00 | 59.00 | 92.00 | 90.00 | 101.00 | 99.00 | 66.00 | 68.00 | 98.00 | 100.00 | 91.00 | 93.00 |  |
|  | NO.17 | 88.00 | 92.00 | 111.00 | 109.00 | 118.00 | 116.00 | 96.00 | 94.00 | 124.00 | 126.00 | 109.00 | 107.00 |  |
|  | NO.18 | 79.00 | 77.00 | 105.00 | 107.00 | 112.00 | 114.00 | 89.00 | 91.00 | 111.00 | 113.00 | 102.00 | 104.00 |  |
|  | NO.19 | 87.00 | 83.00 | 100.00 | 102.00 | 123.00 | 121.00 | 95.00 | 93.00 | 108.00 | 106.00 | 111.00 | 109.00 |  |
|  | NO.20 | 76.00 | 74.00 | 121.00 | 119.00 | 114.00 | 112.00 | 85.00 | 83.00 | 104.00 | 106.00 | 95.00 | 97.00 |  |
|  | NO.21 | 72.00 | 74.00 | 121.00 | 119.00 | 110.00 | 112.00 | 82.00 | 84.00 | 124.00 | 126.00 | 101.00 | 103.00 |  |
|  | NO.22 | 72.00 | 69.00 | 101.00 | 103.00 | 110.00 | 108.00 | 83.00 | 85.00 | 91.00 | 93.00 | 99.00 | 100.00 |  |
| ICC | | 0.946 | | 0.983 | | 0.989 | | 0.979 | | 0.986 | | 0.979 | | |
